# Editorial: Methods in artificial intelligence for dementia 2024

**DOI:** 10.3389/frdem.2024.1444825

**Published:** 2024-07-01

**Authors:** Roozbeh Sadeghian, Fasih Haider, Kathleen Fraser, Shinya Tasaki, Graciela Muniz-Terrera

**Affiliations:** ^1^Department of Data Analytics and Data Science, Harrisburg University of Science and Technology, Harrisburg, PA, United States; ^2^School of Engineering, The University of Edinburgh, Edinburgh, United Kingdom; ^3^National Research Council Canada, Ottawa, ON, Canada; ^4^Rush Alzheimer's Disease Center, Rush University Medical Center, Chicago, IL, United States; ^5^Heritage College of Osteopathic Medicine, Ohio University, Athens, OH, United States; ^6^Centre for Dementia Prevention, University of Edinburgh, Edinburgh, United Kingdom

**Keywords:** digital biomarkers, machine learning, dementia, active living, cognitive impairment, intervention

Dementia refers to a group of neurodegenerative diseases that cause a gradual decline in cognitive functions, affecting memory, thinking, decision-making, language, and motor skills. These symptoms worsen over time, impacting both patients and their caregivers (American Psychiatric Association, 2000). The main causes of dementia include Alzheimer's Disease (which accounts for about 50% of cases), cerebrovascular disease (25%), Lewy body disease (15%), and other conditions like Parkinson's and frontotemporal dementia (5%) (Burns and Iliffe, 2009). The risk of developing dementia increases with age, and as the population of older adults is projected to grow significantly by 2050, the societal impact of dementia care is expected to rise (World Health Organization, 2013).

In 2015, there were about 47.5 million dementia cases worldwide, with new cases annually ranging from 10 to 15 per 1,000 people, most of which are due to Alzheimer's Disease. The average life expectancy after a dementia diagnosis is around 7 years (World Health Organization, 2015). Due to the growing global challenge, there is significant investment in preventing and detecting dementia early. Researchers are seeking cost-effective and scalable methods to identify dementia in its early stages, from subtle signs like subjective memory loss to more severe forms like mild cognitive impairment and Alzheimer's dementia.

This Research Topic aims to highlight the latest experimental techniques and methods of Artificial Intelligence used to investigate fundamental questions in dementia research, from risk factor and biomarker identification to genetics and dementia care. In total, five papers have been accepted on this topic and the findings are summarized below.

Gregory et al. in the paper titled “*Remote data collection speech analysis in people at risk for Alzheimer's disease dementia: usability and acceptability results*” explored the feasibility of phone-based cognitive testing in people at risk for Alzheimer's disease. The study involved 68 participants who had baseline and 3-month follow-up measures. The first test was human-delivered, and the follow-up one was delivered using an automated phonebot. Results showed a high participant retention rate and minimal technical difficulties. The majority of the participants reported ease and comfort using the technology. It was demonstrated that phone-based cognitive assessments through these applications are not only feasible but also acceptable for midlife-to-older adults at risk for AD in the UK, which will have wider applications in remote clinical trials (Gregory et al.).

In the paper “*Alzheimer's disease detection using data fusion with a deep supervised encoder*”, Trinh et al. conducted a large-scale study to determine the most effective method for combining multimodal data to improve Alzheimer's diagnosis. They combined clinical and behavioral information, MRI, and CSF biomarkers in multiple analysis frameworks, ultimately testing over 80 combinations of machine learning classifiers, dimensionality reduction techniques, and data fusion strategies. Their work demonstrates that combining information from multiple modalities can lead to higher diagnostic accuracy while highlighting the importance of appropriately fusing the data, with dimensionality-reduction having a significant impact on performance. The authors also introduce a new method of dimensionality reduction, called *supervised encoder*, which will aid in the future development of computational methods for Alzheimer's disease screening and diagnosis (Trinh et al.).

It is common practice to train everyday voice assistants (EVAs) on large datasets to communicate effectively with the average user. However, these systems are not adapted for people with conditions like dementia, where a slower speech rate and/or long pauses could lead to incorrect clarification requests (CRs). To address this problem, Addlesee and Eshghi explore how to make EVAs more accessible for individuals with dementia by creating interruption recovery pipelines (IRPs). To accomplish this, the authors generated two corpora for evaluation using SPARQL (i.e., SLUICE: a corpus of interrupted questions) and an interrupted AMR corpus. The proposed IRPs are compared against knowledge base question answering (KBQA) and AMR-parsing baselines. They showed that that CRs are effective, and with the SLUICE-CR corpora, the large language models (LLMs) were able to generate appropriate context-dependent CRs. A limitation of this work is its evaluation through user studies (Addlesee and Eshghi).

Orimaye and Schmidtke developed an integrated predictive model (IPM) in the paper called “*Combining artificial neural networks and marginal structural model to predict the progression from depression to Alzheimer's disease*”. In this paper, the authors reported a combined Marginal Structural Model (MSM) and Multi-Layer Perceptron (MLP) Neural Network that is designed to improve prediction for the progression to Alzheimer's disease (AD) in patients stemming from major depressive disorder (MDD). Also, it is shown that the model had a greater utility improvement with the potential to help these patients in early detection and customizing treatment strategies for this high-risk population. An integrated model showed higher predictive performance and better clinical relevance. Therefore, further studies with the potential for utility improvements in early detection and intervention strategies of patients at high risk of progression from MDD to AD should be pursued. The current study discusses the robustness of the IPM through sensitivity analyses and draws inferences in terms of savings in health care costs and patient outcomes (Orimaye and Schmidtke).

Treder et al. explored the potential of large language models (LLMs) in various aspects of dementia research, diagnosis, treatment, and management. In clinical settings, LLMs can be used to summarize medical records, assist healthcare professionals with tasks such as writing clinical reports, and extract relevant information from patient-physician conversations. In research, LLMs are being investigated as tools for predicting the progression of dementia using clinical notes and patient speech. In the management of dementia, potential applications include use in companionship, information retrieval, therapy support, and reading, writing, and navigation assistance. To assess the effectiveness of LLMs in dementia management, a questionnaire administered to both people with dementia (PwD) and their caregivers revealed that all application scenarios were rated as moderately useful to useful. Importantly, both groups prioritized agency over privacy and usability in LLM-based applications. The authors then discussed the technical and design considerations needed to implement LLM-based apps for dementia. The study outlines future research focuses for LLMs, including personalizing LLMs, exploring multimodal LLMs, and integrating LLMs into robotics, all while considering ethical implications and ensuring that the technology meets the diverse needs and preferences of people with dementia (Treder et al.).

The studies presented above are state-of-the-art in dementia research and demonstrate the use of machine learning in addressing the global challenge of dementia.

## Author contributions

RS: Writing – original draft, Writing – review & editing. FH: Writing – original draft, Writing – review & editing. KF: Writing – original draft, Writing – review & editing. ST: Writing – original draft, Writing – review & editing. GM-T: Writing – original draft, Writing – review & editing.

## References

[B1] American Psychiatric Association (2000). “Delirium, dementia, and amnestic and other cognitive disorders,” in Diagnostic and Statistical Manual of Mental Disorders, Text Revision (DSM-IV-TR), chap. 2, ed. M. B. First (Washington, DC).

[B2] Burns A. Iliffe S. (2009). Dementia. BMJ 338:b75. 10.1136/bmj.b75 19196746

[B3] World Health Organization (2013). Mental Health Action Plan 2013-2020. WHO Library Cataloguing-in-Publication Data (Geneva), 1–44.

[B4] World Health Organization (2015). First WHO Ministerial Conference on Global Action Against Dementia: Meeting Report. WHO Library Cataloguing-in-Publication Data (Geneva), 1–76.

